# Understanding the Cognitive Demands, Skills, and Assessment Approaches for Endotracheal Intubation: Cognitive Task Analysis

**DOI:** 10.2196/34522

**Published:** 2022-04-21

**Authors:** Taylor Kunkes, Basiel Makled, Jack Norfleet, Steven Schwaitzberg, Lora Cavuoto

**Affiliations:** 1 Department of Industrial and Systems Engineering University at Buffalo State University of New York Buffalo, NY United States; 2 U.S. Army Combat Capabilities Development Command - Soldier Center Orlando, FL United States; 3 Department of Surgery University at Buffalo State University of New York Buffalo, NY United States

**Keywords:** knowledge elicitation, knowledge acquisition, medical simulation, medical training, medical assessment, critical care, cognitive task analysis, qualitative methods, qualitative, endotracheal intubation, preoperative, training, health care professional, medical education, cognitive skill

## Abstract

**Background:**

Proper airway management is an essential skill for hospital personnel and rescue services to learn, as it is a priority for the care of patients who are critically ill. It is essential that providers be properly trained and competent in performing endotracheal intubation (ETI), a widely used technique for airway management. Several metrics have been created to measure competence in the ETI procedure. However, there is still a need to improve ETI training and evaluation, including a focus on collaborative research across medical specialties, to establish greater competence-based training and assessments. Training and evaluating ETI should also incorporate modern, evidence-based procedural training methodologies.

**Objective:**

This study aims to use the cognitive task analysis (CTA) framework to identify the cognitive demands and skills needed to proficiently perform a task, elucidate differences between novice and expert performance, and provide an understanding of the workload associated with a task. The CTA framework was applied to ETI to capture a broad view of task and training requirements from the perspective of multiple medical specialties.

**Methods:**

A CTA interview was developed based on previous research into the tasks and evaluation methods of ETI. A total of 6 experts from across multiple medical specialties were interviewed to capture the cognitive skills required to complete this task. Interviews were coded for main themes, subthemes in each category, and differences among specialties. These findings were compiled into a skills tree to identify the training needs and cognitive requirements of each task.

**Results:**

The CTA revealed that consistency in equipment setup and planning, through talk or think-aloud methods, is critical to successfully mastering ETI. These factors allow the providers to avoid errors due to patient characteristics and environmental factors. Variation among specialties derived primarily from the environment in which ETI is performed, subsequent treatment plans, and available resources. Anesthesiology typically represented the most ideal cases with a large potential for training, whereas paramedics faced the greatest number of constraints based on the environment and available equipment.

**Conclusions:**

Although the skills tree cannot perfectly capture the complexity and detail of all potential cases, it provided insight into the nuanced skills and training techniques used to prepare novices for the variability they may find in practice. Importantly, the CTA identified ways in which challenges faced by novices may be overcome and how this training can be applied to future cases. By making these implicit skills and points of variation explicit, they can be better translated into teachable details. These findings are consistent with previous studies looking at developing improved assessment metrics for ETI and expanding upon their work by delving into methods of feedback and strategies to assist novices.

## Introduction

### Background

Proper airway management is an essential skill for hospital personnel and rescue services to learn [1-4]; it is the first priority in the management of patients who are critically ill [5]. Airway management includes a set of guidelines and clinical procedures performed to maintain or restore a patient’s airflow to and from the lung [2,5-9]. Endotracheal intubation (ETI) is a widely used technique for airway management in which a laryngoscope is used to obtain a view of the patient’s vocal cords to pass an endotracheal tube into the trachea to facilitate lung ventilation [5,7,10].

ETI is a lifesaving but complicated procedure. The success rate of prehospital intubation is approximately 69.8% and may be as low as 45% [11,12]. Failure to properly ventilate contributes to poor patient outcomes in patients with trauma [6,13,14] and may lead to life-threatening complications, such as hemorrhage, obstruction, tension pneumothorax, or fatality [9,11,13-16]. The risk of an adverse event increases with each subsequent intubation attempt, which can cause tissue damage, edema, or bleeding [9,11,16,17]. Therefore, it is critical that providers be properly trained and competent in performing this procedure. The difficulty of the procedure is determined by patient characteristics and unanticipated events [7,13]. These may include patient weight, atypical airway physiology, any unanticipated obstructions, hemodynamic instability, or compounding injury, which may necessitate cervical spine protection [7,10,11,14].

Despite it being a critical skill in airway management, there is variation in the method of training and way in which to measure competency [18]. Current training methods typically include practicing on cadavers or mannequins and in airway management rotations in clinical settings with live patients [5,9,17,19]. Airway management rotations typically begin by watching in the operating room, where intubation is more routine and easier, and moving to units within the hospital in which intubation is increasingly difficult, such as the intensive care unit [10,19]. However, due to variation in patient anatomy and the need for patient safety, medical residents do not always have the opportunity to practice difficult intubations on live patients [9]. Thus, mannequins and simulators are used instead. Mannequins have been shown to have no direct risk to patients, allow for repeated practice, and act as a bridge between classroom instruction and practical application [10,19].

Although the Accreditation Council for Graduate Medical Education (ACGME) mandates competency in ETI, there is still room for interpretation as to how to evaluate that competency [19]; these measures differ on the number of required procedures, the type of intubation experiences, and the nature of the training program itself. Several metrics have been developed to measure competence in the ETI procedure [12,17,19,20]. Binary item checklists are the most commonly used evaluation metric. However, these provide a summative, rather than formative, assessment of trainee skill [10,12,19]. A recent review of training methods by Brown et al [19] calls for a change to improve ETI training and evaluation. These include a focus on multispecialty research to establish criteria in airway management and establish training collaboration, longitudinal competence-based assessments rather than quantitative assessments, and the incorporation of modern, evidence-based procedural training methodologies [19].

Cognitive task analysis (CTA) is a framework from human factors engineering developed to identify cognitive demands and skills needed to proficiently perform a task; it seeks to describe performance differences between novices and experts and provides an understanding of the workload associated with a task [20]. Methods of CTA typically involve observation and interviews to elicit both explicit and implicit knowledge about a task that may be omitted during free recall [21,22]. Unlike hierarchical task analysis (HTA), which seeks to describe the procedure in terms of sequential tasks and subtasks required to complete a goal [23], CTA seeks to understand and characterize the procedural knowledge of experts to inform tool design and novice informational requirements and to understand potential errors to avoid or correct them [20,22]. An additional advantage of CTA is that it may be conducted with a limited number of subject-matter experts. Although there is little consensus as to exactly how many experts are needed to achieve the greatest knowledge elicitation, it is recommended to interview experts only until enough information is collected [24,25]. Owing to diminishing returns, wherein the amount of new information collected decreases with each new expert interviewed, an ideal number of participants is typically between 3 and 5 participants [24,25].

### Objectives

This study seeks to apply the CTA framework toward the training of ETI. Findings from the CTA would highlight the learning needs of novices toward the development of task expertise and problem solving in adverse events. This work will focus on integrating perspectives from multiple specialties including anesthesiology, emergency medicine, and paramedicine to capture a broad view of task and training requirements. Finally, the results of the CTA will be used to inform a set of skills required to inform a more objective evaluation method for ETI.

## Methods

### Hierarchical Task Analysis

Before conducting the CTA, an HTA was completed to identify the key tasks that occur before, during, and immediately after ETI using direct laryngoscopy. An HTA breaks down the procedure into specific tasks and subtasks from the preparation of equipment and positioning the patient to securing the endotracheal tube in place once it has been inserted. The HTA was completed based on a review of previous studies into assessment of ETI [6,10,26] and gathering information from sources that included web-based videos, reference texts, and observation. Both Ryason et al [10] and Hart et al [26] described the validation of metrics for ETI assessment. These metric tools were used to create an HTA for this study. The HTA started with the following main tasks: (1) initiating the procedure and positioning the patient, (2) inserting the direct laryngoscopy blade, (3) achieving the optimal laryngeal view, (4) inserting the endotracheal tube, (5) verifying tube placement, and (6) securing the endotracheal tube. Subtasks were not explicitly described but were used to inform the creation of an interview guide.

### CTA Interview Development

Interview questions were developed to capture a broad array of the steps required to complete an ETI and the necessary cognitive requirements to complete those activities. These included the specific goals of each step (and substep), the main challenges faced by novices and strategies used by experts to overcome these challenges, methods of feedback, and current measures of proficiency. The questions were organized by subtask. The HTA and previous assessment tools served as a guide for systematically developing CTA questions for each task in the ETI procedure.

The interviews were semistructured, beginning with broad questions about the task (eg, “What are the main goals and priorities during this step?”) with subsequent questions based on the response for clarification or to increase the interviewer’s understanding (eg, “Can you explain the difference between the use of a stylet versus a bougie as an assistance device?”). Further questions expanded on the differences between performing ETI across varying specialties (eg, anesthesiology in the operating room, emergency medicine in a hospital emergency department, or paramedicine in someone’s home). Additional questions were used to help discern implicit aspects of the cognitive processes of the procedure, such as decisions involved and methods to determine proficiency (eg, “What visual or tactile feedback do you use to determine the [laryngoscopy] blade is positioned correctly?”). Example questions from the interviews of ETI are provided in Textbox 1.

**Exemplar questions from the cognitive task analysis interview with endotracheal intubation experts.**

**Cognitive task analysis interview questions**

- Describe the sequence of actions necessary to complete this step.
- What are the main goals and priorities during this step?
- What tools do you gather to begin? What factors determine this choice?
- What do you look for to determine proficiency in this step?
- In what instances might you need to use additional strategies to gain better visualization or understanding of landmarks?
- What steps do novices seem to have problems learning or performing?
- What is the proper grip to hold the laryngoscopy blade?
- What visual or tactile feedback do you use to determine the blade is positioned correctly or needs to be adjusted?
- What is the optimal laryngeal view?
- What feedback measures do you use to determine correct placement of the endotracheal tube?
- What methods do you use to test the security of the endotracheal tube?
- What problems should an expert be able to solve if they have mastered endotracheal intubation?
- At what point do you intervene if a task is being performed incorrectly? What does this intervention look like?

**Follow-up questions to prompt further discussion**

- What alternative strategies could you use?
- What precautions or preventative measures do you use to overcome any risks?
- What skills are involved in completing this step?

Interviews were conducted using Zoom (Zoom Video Communications, Inc) video conferencing and in-person observation of the procedure on mannequins. Data were recorded through handwritten notes and audio and video recordings of the interviews. Zoom conferencing provided a transcription of each recording, which was reviewed against the audio recording for accuracy before the analysis. Each interview lasted approximately 60 minutes. Participants were briefed on the study objectives before meeting with the researcher conducting the interview.

### Ethical Approval

The study was approved by the institutional review board at the University at Buffalo (STUDY00004879) and the Army Human Research Protection Office (ARL 21-007).

### Participants

Interviews were conducted with 6 participants from multiple specialties and work environments to gain diverse perspectives on the procedure. The participants were 2 anesthesiologists, 2 emergency medicine physicians, and 2 paramedics. The participants were from Florida, New York, and Ontario, Canada. The participants were all considered experts in their field, and each participant serves as an instructor in their respective setting.

These medical specialties were chosen based on their job descriptions and responsibilities and the nature of medical procedures they can perform. Anesthesiologists complete 12 to 14 years of formal education, including 12,000 to 16,000 hours of clinical training [27]. They are required to evaluate, monitor, and supervise patient care before, during, and after surgery and may assist in pain management [27,28].

Emergency medicine physicians complete 11 to 12 years of formal education, of which 3 to 4 years are residency in an emergency medicine program. Emergency medicine physicians must stabilize and treat patients until emergency care is no longer required. This includes completing a patient assessment, diagnosing illness or injuries, ordering appropriate medical exams and treatments, creating and communicating postemergency plans with the patient and other providers as necessary, and facilitating the patient transition to their home or living environment or to another department in the hospital [29,30]. Although education requirements may differ, the job responsibilities among emergency medicine physicians in the United States and Canada are comparable [30].

Finally, paramedics receive the least amount of formal school-based training. Paramedics must hold an emergency medical technician–basic (EMT-B) certification before applying for a paramedic program. The EMT-B program typically requires 150 to 180 hours of training. Paramedics must complete an additional 1200 to 1800 hours of training in a nationally accredited program and pass both written and practical examinations [31]. Paramedics are responsible for responding to emergency calls for medical assistance; must assess, triage, and treat patient physical and psychological needs; and facilitate referrals to a higher level of medical care when necessary [31]. Unlike an EMT-B, who can perform basic life support, a paramedic may perform more complex medical procedures and administer medication [31].

### Analysis

A thematic analysis of the qualitative data from the interviews was conducted primarily by a single researcher (TK), a PhD student in human factors. Deductive coding was used to create the following high-level categories based on the interview guide: main goals, challenges, methods of feedback, strategies, and measures of proficiency. A deeper analysis using inductive coding was conducted to identify further themes related to the cognitive aspects of ETI. These themes were identified and organized within each high-level category for each task of the ETI procedure. Owing to variation in cognitive skills required across specialties, a separate analytic framework was developed to delineate competencies among the specialties examined in this study: anesthesiology, emergency medicine, and paramedicine. This allowed for further analysis of the differences between ideal and complicated cases; for example, in a hospital environment, the patient is typically in an accessible location, whereas outside the hospital, the patient may need to be moved to a location where the provider can perform ETI. The themes and subthemes were incorporated into a skills tree depicting the various cognitive skills and insights in each step of the procedure; this skills tree can then be used to develop training tools or provide greater insight into assessment metrics.

## Results

### Overview

A total of 6 participants were interviewed and observed. Of these 6 participants, 5 (83%) were interviewed over Zoom video conferencing, and 1 (17%) was interviewed in person. In addition, 33% (2/6) of the participants provided a demonstration of the ETI on a mannequin. Interviews were conducted by a single researcher (TK). Of the 6 participants, 2 (33%) were anesthesiologists, 2 (33%) were emergency medicine physicians, and 2 (33%) were paramedics. The participants had an average of 17 years of experience: anesthesiologists 18 years, emergency medicine physicians 17 years, and paramedics 16 years. All participants held educational roles in their respective facilities and were considered experts in their field.

The interviews elicited the cognitive demands and procedural skills required to complete ETI from the perspectives of practitioners across multiple specialties. These interviews further highlighted the training requirements, stumbling points, and measures of proficiency that novices experience when performing this procedure. Furthermore, the interviews showed the differences and similarities in cognitive requirements when performing ETI within different medical specialties and environments. The findings from these interviews were categorized according to each step of the ETI procedure and emergent themes, as shown in Table 1.

Five broad, high-level categories were identified for each step of the ETI procedure: main goals, challenges, methods of feedback, strategies to assist, and measures of proficiency. Emergent themes within each of the above categories were mapped to the various steps of the ETI procedure. This represents the interconnected nature of each category of cognitive insights, as they relate to a specific task or subtask within the procedure. For example, under the “Insertion of the Endotracheal Tube” task, the main goal is to successfully insert the tube. However, deviations in the patient’s anatomy, noted under challenges, may arise when the airway is smaller than anticipated, and it is difficult to actually pass the endotracheal tube past the vocal cords. A strategy that could be used is to advance a bougie past the vocal cords first, as it has a smaller diameter, and then insert the endotracheal tube over the bougie.

The content in each theme within the skills tree table can be elaborated based on the complexity of each case. However, the key insights represented here provide a level of detail sufficient to the design of new cross-specialty training methods and assessment tools.

**Table 1:** Skills tree for endotracheal intubation.

	Main goals	Challenges	Methods of feedback	Strategies to assist	Measures of proficiency
Overall procedure	Consistency Timing (preoxygenation [5 minutes], equipment setup, and procedure [30-60 seconds])	Provider-related: confidence in ability to perform a procedure and to lead medical team; maintain composure Patient-related: atypical anatomy or unanticipated events, patient’s medical conditions, and airway difficulty Procedure-related: safety of the patient and provider and type of intubation (rapid-sequence or awake)	Talk-aloud method (prior to real case, during real case, and contingency planning for simulated case) Mannequins and simulators Video laryngoscope	Medical team (reassurance of expert to provide feedback and to take over procedure; other team members to provide assistance and to monitor patient vitals) Procedure talk-through method (prior to real case and during real case)	Planning for procedure (clear and concise, contingency for real or potential problems, and next steps [what comes after ETI^a^]) Consistency (in each step) Timing Communication with medical team Number of successful intubations (on a mannequin and person) Lack of trauma to lips, teeth, or airway tissues Accreditation Council for Graduate Medical Education Requirements
Step 1: preparation and positioning	Ventilate and preoxygenate the patient Ensure functionality of equipment (medical equipment and medication) The patient is positioned correctly (supine, sniffing position and appropriate height for provider)	Consistency (equipment is set up in the same manner each time and all equipment and materials are available) Timing (insufficient preoxygenation) Patient characteristics (facial hair, weight, anatomical challenges [eg, jaw size and neck mobility], and other injuries)	Patient body position (supine, sniffing position and height of surface on which patient is laying) Suitability of equipment (chosen tools are appropriate for the patient) Indication for intubation	Consistency Use of additional equipment (to change equipment if needed and to reposition the patient)	Correct indication for intubation Consistency over multiple attempts The patient is positioned correctly Suitability of equipment (appropriate equipment sizes and medication)
Step 2: inserting the direct laryngoscopy blade	Timing (approximately 30 seconds) Visualization (keep airway clear to see while inserting laryngoscopy blade)	Motor skills (rocking blade back instead of lifting, not sweeping tongue to the left, lifting blade too early, using excessive force to lift blade) Patient-related factors (abnormal/atypical anatomy and obstructed view) Movement speed (move too quickly, leading to additional challenges)	Field of vision (can provider see vs can they not see)	Talk-aloud method (slows down procedure to allow the provider to visualize and provides the instructor an opportunity to understand trainee’s view) Make a change (to equipment and technique)	Talk-aloud method Blade position Pre-assessment determinations (airway difficulty determines timing and may influence repositioning and number of reattempts that are reasonable) Lack of trauma to patient
Step 3: achieving the optimal view	See tracheal opening (achieve a view in which the provider can pass the endotracheal tube through the vocal cords)	Motor skill (blade in correct position and use of force rather than fine motor control) Obstructed view (due to abnormal or atypical anatomy)	Talk-aloud method (provides the instructor an opportunity to understand the trainee’s view) Video review (if available)	Reposition the patient (body supports, pull on the right side of the mouth, and the BURP^b^ technique) Review patient history (maintain composure) Change equipment SALAD^c^ technique	Verbal review (of technique and landmarks) Timing (5-10 seconds)
Step 4: inserting the endotracheal tube	Tube inserted correctly (into the trachea and at the correct depth) Minimize trauma to airway tissues	Incorrect positioning of endotracheal tube (into the esophagus, into only 1 bronchus, and too shallow) Premature removal of equipment	Stopping criteria (depth mark on tube at the teeth or lips and can tube continue to advance [likely in esophagus] or not [likely in trachea]) Tactile sensation (feel of tracheal rings against bougie)	Use of assistance device (stylet and bougie) Tube adjustment (hold toward the end of the tube rather than hold the tube near the lips and bend the tube using a stylet into a hockey stick shape [≤45° angle]) Change equipment (stylet to bougie; tube dimension) SALAD technique	Depth of tube Number of attempts to insert tube Timing
Step 5: verifying endotracheal tube placement	Established airflow to the lungs (endotracheal tube is in the correct position for appropriate oxygenation)	Verification methods (available tools for verification and objectivity of verification methods) Position of endotracheal tube (in the esophagus rather than the trachea, in 1 bronchus rather than 2, and not far enough into the trachea)	Visual methods (visualization of tube going through cords and tube changing color due to mist) Auditory methods (auscultation of breath sounds) Device-assisted methods (end-tidal CO_2_, chest x-ray, and ultrasound) Patient vital signs	Readjustment of tube (to a different depth) Reattempt to insert endotracheal tube	Patient has airflow to both lungs
Step 6: securing the endotracheal tube	Stability of endotracheal tube placement (ensure endotracheal tube will not move after placement)	Available tools (tape and securement device) Securing tube to another tube (eg, to NG^d^ tube) Failure to plan for postintubation activities	Tug test (lightly pull on the endotracheal tube to test security) Visual examination of tape or tube securement device	Avoid unnecessary movement of tube Tug test (excessive force during the tug test may extubate the patient prematurely)	Endotracheal tube does not move or come out

^a^ETI: endotracheal intubation.

^b^BURP: backwards, upwards, right, pressure.

^c^SALAD: Suction-Assisted Laryngoscopy Airway Decontamination.

^d^NG: nasogastric.

### Main Goals

Main goals refer to the overarching objective of each task within ETI. Achieving these goals determines if that task is considered successful and if the practitioner is ready to move to the next step. Themes in the main goals category provide a basis for the evaluation of trainee success. Achieving these goals, in conjunction with feedback received, as described in the “Methods of Feedback” section, allows the trainee to determine if they should proceed to the next step or make a corrective action at the current step.

The degree to which each goal is achieved may vary owing to differences in task complexity, patient characteristics, and the environment in which the ETI is being performed. For example, in achieving the optimal view, the main goal is to achieve a view of the patient’s soft palate and tracheal opening through which the provider can pass the endotracheal tube. A provider mentioned:

...there are four views you can get...you can either see the vocal cords completely...or you can see only a very small part of the opening.

In an ideal case, the provider would be able to see the epiglottis and the vocal cords in the center of the field of vision.

Although some variation results from patient or environmental characteristics, there may also be acceptable deviation in provider performance. This can be clearly seen in the first step of the procedure: positioning the patient. The patient should always be in a supine and sniffing position by which the patient’s ears are at the level of their shoulders and it looks as if the patient is sniffing flowers. However, the amount of padding or the angle at which the patient is positioned (eg, in reverse Trendelenburg) are variable.

### Challenges

Challenges are instances in which novices typically experience difficulty when performing ETI. These may be typical or routine obstacles that may occur during the procedure, unanticipated events, or barriers that arise from the provider’s lack of experience. Typical challenges include anatomy- and physiology-related obstacles. For example, the patient may have a small mouth and jaw. As a result, the provider may need to use a smaller laryngoscopy blade or may experience difficulty in maneuvering the blade into position without causing damage to the patient’s teeth or airway tissues. Problems or experiences that cannot be controlled or accounted for before the procedure are considered unanticipated events. The most commonly noted unanticipated event was an obstructed airway that was undetected in a preintubation airway assessment, such as a subglottic stenosis.

Challenges resulting from the provider’s lack of experience include both behavioral- and skill-related barriers to ETI. The provider’s confidence, or lack thereof, in their ability was frequently cited as a behavioral barrier. A provider noted:

...an older medic who’s done this a bunch of times may spend longer...and know they’re almost where they need to be whereas novices may get in there and spend 15 seconds...and give up on that.

Another provider discussed the provider’s confidence in leading the medical team; if the provider is not confident in their ability, the rest of the medical team may also feel anxious. Skill-related barriers are those related to the motor skill necessary to perform ETI, such as lifting the laryngoscopy blade to achieve the optimal view rather than rocking the blade back into the patient’s teeth.

Addressing these challenges during training allows novices to develop decision-making strategies for anticipated and unanticipated barriers to ETI. This supports the provider in assessing the trainee’s ability to plan for future cases. It also provides insight into cognitive and motor skills where additional training may be required. The specific challenges a novice faces may influence what additional strategies are used to assist the provider in completing the procedure. Finally, by addressing these challenges explicitly during training, the novice may feel a greater sense of accomplishment when overcoming these hurdles and thus boost their confidence.

### Methods of Feedback

Methods of feedback refer to the external elements used by the provider to determine if they are performing the step correctly or if any adjustments are required. Feedback may be visual, tactile, or auditory. Visual methods of feedback include those that the provider can see. For example, when positioning the patient, the provider must also consider the height of the bed. The patient should be at a height “where [the provider’s] elbows bend at about 90°.” Some elements, such as this, are visible to both the novice performing the ETI and the trainer observing. Other visual elements are only seen by the novice, such as if the provider can see the patient’s vocal cords or not. In this case, additional strategies to assist performance may also be necessary.

Auditory methods of feedback primarily include talk-through protocols. During training, and before real cases, novices may discuss the procedure and potential problems that may arise. This allows the novice to verbally demonstrate their knowledge of the procedure and problem-solving capabilities. Verbal protocols also provide the trainer with information on what the novice is seeing during steps where there is limited visual feedback, such as when attempting to achieve the optimal view. Auditory methods also include the use of auscultation to verify placement of the endotracheal tube. Using a stethoscope, the physician listens for breath sounds over the stomach and both lungs to ensure that there is appropriate airflow.

Finally, tactile methods of feedback encompass those that the novice can feel. For example, providers can use stopping criteria when inserting the endotracheal tube. If the endotracheal tube can continue to advance, it is an indicator that it is likely in the esophagus and a second attempt is required; however, if the endotracheal tube stops and cannot be advanced further, it is likely in the trachea.

Incorporation of these methods of feedback into training provides novices with additional tools by which to evaluate and adjust their performance. Not all methods may be necessary in each case owing to natural variation in complexity; however, these methods are translatable to all cases. Furthermore, these methods of feedback can be incorporated into training as evaluation criteria for each task within the ETI procedure.

### Strategies to Assist

Strategies to assist are tools used by the provider to avoid or overcome complications presented during the ETI procedure. Commonly, these are used in response to a presented challenge or in conjunction with methods of feedback to adjust performance. The most commonly presented strategies were for the provider to make a change in how they are approaching the task. For example, when inserting the laryngoscopy blade, switching from a Miller blade to a MacIntosh blade may allow the provider to gain better visualization for later steps. Other reported physical strategies included the use of additional equipment, such as padding to reposition the patient or portable suction to remove blood or emesis obstructing the view of the airway. Additional strategies include talk-through protocols, which force the novice to slow down and take stock of what they are seeing and doing and how consistently the novice performs the procedure over multiple attempts. These strategies are cultivated by experts in order to assist trainees in developing their motor skills and build confidence in their ability. As such, it is critical to identify these strategies as requisite tools to successfully completing ETI.

### Measures of Proficiency

Measures of proficiency refer to the assessment methods used by a trainer to determine if the novice is successfully performing the procedure. These measures of proficiency provide a baseline for evaluation at each step of the procedure. Thus, providers can use these measures to target specific steps for additional training. These also allow for insight into which additional strategies or feedback may be most beneficial to the trainee.

Examples of measures to evaluate motor skill proficiency include observer assessment of patient positioning, laryngoscopy blade position, and the depth measurement mark on the endotracheal tube after it is inserted. In addition, the trainer may use talk-through protocols to determine if the trainee is progressing appropriately during the procedure; for example, the trainee may announce landmarks within the mouth and throat as they are advancing the laryngoscopy blade. Talk-through protocols also provide insight into the trainee’s problem-solving abilities, similar to what might be experienced during medical licensing examinations.

Other measures of assessment include the time taken to complete the ETI, not including preoxygenation, how consistently the provider performs over multiple intubations, and the number of successful intubations performed. Providers reported varying counts of successful intubations for proficiency; a provider from anesthesiology cited that 50 successful intubations would be sufficient to call someone proficient, whereas an emergency medicine physician cited over 100 intubations throughout 5 years of residency. Other providers referenced the ACGME requirements as the standard for measuring proficiency across intubation attempts, with a minimum requirement of 35 successful intubations. The ACGME defines 5 levels of competency in airway management. At level 1, novices are expected to describe the airway anatomy. By level 5, the novice should be able to teach airway management skills to other health care providers. Although the ACGME does not have specific evaluation metrics, the suggested methods of evaluation include airway management assessment cards, checklists, procedures logs, and simulations.

### Themes by Specialty

The participants in this study were from various specialties. As such, differences were found in the learning requirements, strategies to assist, and methods of feedback and measures of proficiency used by trainers. These differences are listed in Table 2. The table distinguishes the following specialties: anesthesiology, emergency medicine, and paramedicine. Overall, there are a few key differences between the specialties. Variation among specialties primarily derived from the environment in which ETI is performed, subsequent treatment plans, and available resources.

Anesthesiology typically represented the most ideal cases for intubation; providers cited anesthesiology as the first specialty students are placed during airway management rotations. In anesthesiology, ETI is performed in an operating room with a full staff to assist. Presurgical assessments and patient review ensures the provider can select the appropriate equipment and medication for that patient. In emergency medicine, the patient may still be in the hospital, but there may not be time to conduct a full airway assessment or patient history before performing ETI. Thus, it may be more difficult to determine the correct equipment sizes or medication.

Paramedics are limited by the equipment and personnel available on a particular emergency call. In addition, a paramedicine trainer noted “if [the patient is] in a very small or a very dimly lit area, I will immediately try to see if we can move the patient.” The provider must continue to perform basic life support while moving the patient to a new location to begin the ETI procedure without the assistance of a full medical team.

Finally, the postintubation plan is different among specialties. In anesthesiology, the patient is about to undergo a surgical procedure; therefore, the postintubation plan is to monitor the patient throughout the surgery. Likewise, paramedics have a known postintubation plan: transport the patient to a medical facility for further care. However, in emergency medicine, the next steps are not always obvious to novices. An emergency medicine physician noted “one thing [novices] forget about is having sedation ready for afterwards.” Regardless of what is required after intubation, it is critical to provide sedation so that the patient does not attempt to remove the tube prematurely and without assistance.

**Table 2:** Differences of endotracheal intubation among medical specialties.

		Anesthesiology	Emergency medicine	Paramedicine
**Preparation and positioning**				
	Challenges	Patient characteristics: patient undergoes presurgical assessment; typically, most ideal cases and patient characteristics that influence difficulty of ETI^a^ are addressed before procedure Environment: operating room with full staff to assist; easy access to equipment and medication	Patient characteristics: patient may or may not have an airway assessment completed Environment: clinical room with staff to assist; easy access to equipment and medication	Patient characteristics: patient does not have an airway assessment Environment: nonclinical setting; patient may need to be moved from a small space, such as a closet, to a location in which the provider has sufficient room to perform ETI; provider may be hunched over the patient rather than having elbows at 90° angle Equipment: do not use medication, as there is insufficient patient history to administer the correct medication and limited storage in the ambulance
**Inserting the direct laryngoscopy blade**				
	Challenges	Patient-related factors: airway assessment allows provider to account for patient variability (eg, facial hair)	Patient-related factors: airway assessment, if available, allows provider to account for patient variability (eg, facial hair)	Patient-related factors: unable to account for patient variability
	Strategies to assist	Potential for video laryngoscope	Potential for video laryngoscope	Video laryngoscope not always available
**Achieving the optimal view**				
	Strategies to assist	Reposition patient: additional equipment and assistance available to maneuver patient into sufficient position for intubation Suction: readily available	Reposition patient: additional equipment and assistance available to maneuver patient into sufficient position for intubation Suction: readily available	Reposition patient: limited equipment and assistance available to maneuver patient into sufficient position for intubation Suction: portable suction may or may not be available
**Verifying the endotracheal tube**				
	Methods of feedback	Available methods: availability of visual, auditory, and medical devices to verify placement of endotracheal tube	Available methods: availability of visual, auditory, and medical devices to verify placement of endotracheal tube	Available methods: limited verification methods available: end-tidal CO_2_ monitor and visual and auditory methods which may be subject to human error
**Securing the endotracheal tube**				
	Challenges	Postintubation activities: postintubation activity is known and planned for other medical procedures	Postintubation activities: may be a failure to plan for postintubation activities	Postintubation activities: postintubation activity is known and planned for transportation to a medical facility Environment: tube may be moved during transportation

^a^ETI: endotracheal intubation.

## Discussion

### Principal Findings

CTA interviews with experts in ETI discerned the five following cognitive skills and processes necessary to perform and evaluate the procedure: (1) the main goals, (2) challenges faced by novices when performing ETI, (3) methods of feedback by which the individual performing the procedure can gauge success, (4) strategies used by experts to assist performance, and (5) measures of proficiency by which novices are evaluated at each step. The skills tree depicts these various cognitive skills and processes, as well as the implicit subskills and areas of variation, at each step. This allows for the identification of challenges faced by novices and the ways in which these challenges may be overcome. Furthermore, the breakdown of skills by subtask allows these points to be better translated into teachable details.

Interviews with experts in ETI from various specialties assisted in identifying and systematically representing the cognitive processes and skills required to perform ETI tasks. These cognitive processes include the main goals, challenges novices face, methods of feedback, strategies used to assist performance, and measures of proficiency of each step, as defined by previous HTA and assessment metrics [6,10,26]. These insights are critical for the development of training and objective assessment tools. In addition, the CTA highlighted the complexity of the procedure arising from challenges providers may face because of environmental or patient-related variability.

Although the CTA did not capture the complexity and detail of all potential cases, it provided insight into the nuanced skills and training techniques used to prepare novices for the variability they may find in practice. Importantly, the CTA identified ways in which challenges faced by novices may be overcome and how this training can be applied to future cases. For example, the trainee may be asked to verbally dictate their contingency plan for inserting the endotracheal tube should the airway prove to be more difficult. During this process, the trainee could be continuously provided increasingly difficult or complex adverse events to assess their ability to solve problems at each potential pitfall.

The skills tree developed from the CTA interviews highlights the various subskills and areas of variation that are not always easily explicitly taught to novices. For example, patient variability may include neck mobility, other injuries, facial hair, and gender. Characteristics such as neck mobility, jaw size, or the presence of other injuries or medical conditions to monitor are routinely discussed. However, the presence of facial hair or gender may pose unique challenges that are not always explicitly discussed before a case where they become applicable. Facial hair may disrupt the ability of the bag mask to seal properly on the patient’s face, resulting in poor preoxygenation, and women typically have breast tissue, which, depending on the patient’s position, may add additional strain on the neck or chest, which increases the difficulty of the intubation. These subthemes in each step refer to the nuances of skills and maneuvers required to complete each task. By making these implicit themes explicit, they can be better translated into teachable details. These findings are consistent with previous studies looking at developing improved assessment metrics for ETI [10,13,19,26] and expand upon their work by delving into methods of feedback and strategies to assist novices.

Although CTA interviews are advantageous because they require a small number of participants, it is possible with a greater number of participants from all 3 disciplines that a greater number of cognitive processes and strategies would emerge. In addition, a greater number of experts could serve to highlight greater differences between medical specialties and discern additional aspects of training unique to those specialties.

### Conclusions and Future Work

Findings from the CTA as depicted in this paper can only be validated through expert review and through the development of training systems. Future work may focus on the difference in cognitive skills between novices and experts performing ETI in simulated cases, error recognition and repair, and task switching in which cognitive skills developed in learning ETI are applied to a different procedure. The CTA also highlighted instances where cross-specialty training may provide a new dynamic to the training method and provide a wider application for training in various fields, such as military combat medic training.

## Abbreviations

ACGME: Accreditation Council for Graduate Medical Education
CTA: cognitive task analysis
EMT-B: emergency medical technician–basic
ETI: endotracheal intubation
HTA: hierarchical task analysis
